# PEG Spacer Length Substantially Affects Antibody-Based Nanocarrier Targeting of Dendritic Cell Subsets

**DOI:** 10.3390/pharmaceutics14081614

**Published:** 2022-08-02

**Authors:** Maximilian Brückner, Michael Fichter, Richard da Costa Marques, Katharina Landfester, Volker Mailänder

**Affiliations:** 1 Department of Dermatology, University Medical Center of the Johannes Gutenberg University Mainz, Langenbeckstr. 1, 55131 Mainz, Germany; brueckner@mpip-mainz.mpg.de (M.B.); fichter@uni-mainz.de (M.F.); dacostamarques@mpip-mainz.mpg.de (R.d.C.M.); 2 Max Planck Institute for Polymer Research, Ackermannweg 10, 55128 Mainz, Germany; landfester@mpip-mainz.mpg.de

**Keywords:** antibody functionalization, nanoparticles, nanovaccine, dendritic cell targeting, PEG

## Abstract

Successful cell targeting depends on the controlled positioning of cell-type-specific antibodies on the nanocarrier’s (NC) surface. Uncontrolled antibody immobilization results in unintended cell uptake due to Fc-mediated cell interaction. Consequently, precise immobilization of the Fc region towards the nanocarrier surface is needed with the Fab regions staying freely accessible for antigen binding. Moreover, the antibody needs to be a certain distance from the nanocarrier surface, influencing the targeting performance after formation of the biomolecular corona. This can be achieved by using PEG linker molecules. Here we demonstrate cell type-specific targeting for dendritic cells (DC) as cellular key regulators of immune responses. However, to date, dendritic cell targeting experiments using different linker lengths still need to be conducted. Consequently, we focused on the surface modification of nanocarriers with different molecular weight PEG linkers (0.65, 2, and 5 kDa), and their ability to reduce undesired cell uptake, while achieving efficient DC targeting via covalently immobilized antibodies (stealth targeting). Our findings demonstrate that the PEG linker length significantly affects active dendritic cell targeting from cell lines (DC2.4) to primary cells (BMDCs, splenocytic conventional DCs type 1 (cDC1)). While antibody-functionalized nanocarriers with a shorter PEG length (0.65 kDa) showed the best targeting in DC2.4, a longer PEG length (5 kDa) was required to specifically accumulate in BMDCs and splenocytic cDC1. Our study highlights that these crucial aspects must be considered when targeting dendritic cell subsets, which are of great importance in the fields of cancer immunotherapy and vaccine development.

## 1. Introduction

The main goal of smart drug delivery is the targeted nanocarrier transport of drugs, or immunomodulatory molecules, to the organ or cell type of interest without clearance or accumulation in other compartments [1]. Controlling nanocarrier-based delivery is key to the reduction in systemic side effects and to the improvement of pharmacological profiles [2]. Despite many years of research, tumor-targeting nanocarrier systems only achieve tumor accumulation rates with a median of 0.7% of the injected dose at the targeted site [3]. Consequently, nanocarriers applied as nanomedicines have generally failed in clinical trials, with only a few exceptions. All of these nanomedicines have no targeting moiety incorporated into the nanocarrier. These nanocarriers include albumin-stabilized paclitaxel nanoparticles (ABRAXANE^®^), or liposomal formulations of either doxorubicin (DOXIL^®^) or irinotecan (ONIVYDE^®^) [4,5,6]. Nevertheless, nanocarriers inherit a great potential in revolutionizing targeted therapies, as demonstrated just recently by the use of lipid nanoparticle-based COVID-19 mRNA vaccines [7].

Numerous factors influence successful clinical translation into the medical market and must be overcome to learn from and recognize previously failed nanocarrier systems [5,6]. In this field, the nano-bio interface represents a great challenge [6]. In particular, we are talking about the role of the biomolecular corona and its influence, antibody-based nanocarrier targeting [8], and drug release [9]. In addition, many nanocarrier systems are rapidly cleared by the mononuclear phagocyte system (MPS), or the complement system [10,11,12]. In this process, pristine nanocarriers are usually marked and identified by opsonins, such as complement proteins or immunoglobulins, which can bind to opsonin receptors expressed on macrophages leading to nanocarrier phagocytosis [13]. Consequently, the immune system plays a critical role in the recognition and elimination of nanocarriers [14].

Therefore, nanocarriers have been coated with an additional stealth layer that disguises the nanocarriers and prevents their recognition by opsonins, thus reducing phagocytic removal. Among the stealth polymers, poly-(ethylene glycol) (PEG) is the gold standard or benchmark for surface modification of nanocarriers resulting in reduced clearance by the MPS and complementary system [15]. PEG has been shown to increase the hydrodynamic size and water solubility of nanocarrier systems, thereby decreasing their self-aggregation and interaction with blood components of the MPS by means of steric repulsion [16,17]. The use of PEGylated nanocarrier systems are rapidly increasing in the field of nanomedicine, and have now reached clinical trials, or have been licensed by pharmaceutical regulatory authorities [18]. Among the PEGylated drugs or nanocarrier systems, attached PEGs differ in their molecular weight ranging from 2 to 60 kDa [17]. However, these non-targeted nanocarriers demonstrate an improved pharmacokinetic profile compared to the administration of a free payload [19]. However, as mentioned above, the average accumulation at the target site is low in the case of tumor therapy [3].

Consequently, improving selective accumulation of the nanocarrier system requires active targeting ligands, such as surface decoration with antibody molecules. In the field of cancer vaccines and immunotherapy, a nanocarrier-based targeted delivery of tumor-associated antigens and immunomodulators (adjuvants) to dendritic cells (DCs) bears great potential since dendritic cells are professional antigen-presenting cells capable of generating robust antigen-specific anti-tumor immune responses [20]. However, due to the poor success of DC-based anti-tumor vaccines in clinical trials, an efficient targeting of specific DC subsets is considered to be the prerequisite for inducing strong anti-tumor responses [21]. In particular, conventional DCs type 1 (cDC1) inherit the potential to efficiently cross-present exogenous antigens enabling them to induce strong cytotoxic T cell responses against tumor cells [22]. Therefore, delivering cargo to cDC1 via nanocarriers functionalized with antibodies, such as anti-CLEC9A [23] and other targets, is a feasible approach. However, there are several limitations evidenced by the small number of antibody-functionalized nanocarrier formulations currently undergoing clinical trials [24]. Most commonly, in the antibody surface-decoration of nanocarriers a PEG linker is applied to locate the antibody molecule at a certain distance from the nanocarrier surface. Hence, the PEG length can influence the interactions of the attached targeting ligand and the cell receptor of interest [25,26].

As investigated by Cruz et al., a PEG linker length comparison on antigen-loaded and antibody-coated PLGA nanoparticles highlighted the finding that short PEG constructs (2–3 kDa) result in stronger interactions with DCs, inducing higher T cell proliferation when compared to longer PEG constructs (6–20 kDa) [27]. In contrast, Kapadia et al. demonstrated a higher particle uptake and antigen presentation of peptide-decorated hydrogels when using a 5 kDa PEG linker compared to 2 and 10 kDa [28]. Given this complexity, we focused on investigating previously developed antibody-nanocarrier conjugates, precisely targeting DCs both in vitro [29] and in vivo [23], with respect to the influence of the applied PEG linker length (0.65, 2 and 5 kDa PEG). Our investigation focused particularly on the targeting of the CD11c receptor on DCs in immortalized (DC2.4), or primary DCs (BMDCs, splenocytic cDC1s, and the impact of the biomolecular corona. This study delineates the differences in DC targeting between the three PEG linker constructs from cell lines to primary cells, highlighting a greater antibody distance from the nanocarrier surface necessary for targeting DC subsets. In particular we have shown that the ability of nanocarriers to target cDC1 in primary cells required a 5 kDa PEG construct. Furthermore, our biomolecular corona analyses highlight the importance of the nanocarrier–antibody composition.

## 2. Materials and Methods

**Cell culture.** The murine RAW264.7 macrophages (DSMZ, Braunschweig, Germany) were cultured in Dulbecco’s modified eagle medium (DMEM, Thermo Fisher Scientific, Waltham, MA, USA containing 10% fetal bovine serum (FBS), 100 U mL^−1^ penicillin, 100 mg mL^−1^ streptomycin, and 2 mM glutamine (all Thermo Fisher Scientific, Waltham, MA, USA). The murine DC2.4 dendritic cells (Merck, Darmstadt, Germany) were cultured in Iscove’s Modified Dulbecco’s Medium (IMDM, Sigma-Aldrich, St. Louis, MO, USA), supplemented with 5% FBS, 100 U mL^−1^ penicillin, 100 mg mL^−1^ streptomycin, 2 mM glutamine, as well as 1% 2-Mercaptoethanol (100×). Both cell lines were maintained in an incubator at 37 °C and 5% CO_2_ humidity (CO_2_ Incubator C200, Labotect, Rosdorf, Germany). For the passaging of cells, the consumed media was discarded and the cells were briefly washed with 10 mL PBS. RAW264.7 cells were detached at 37 °C with Trypsin-EDTA (Thermo, Germany) and DC2.4 cells at 4 °C with PBS containing 2 mM EDTA for 10 min. The cells were detached from the flask by pipetting 10–20 mL of FBS-containing medium up and down, followed by centrifugation of the cell suspension at 300× *g* for 5 min. The resulting cell pellet was resuspended in medium with FBS and the cell viability was tested with trypan blue (1:1 dilution) using an automated cell counter (TC10, Bio-Rad, Hercules, CA, USA).

**Nanocarrier PEGylation and modification for the attachment of antibodies.** The nanocarriers, namely bionized nanoferrite particles with a hydroxyethyl starch shell (Micromod, Rostock, Germany), were PEGylated to the surface amine groups via NHS chemistry based on the amount of NH_2_ given by the manufacturer (3 nmol mg^−1^). For this purpose, five different bifunctional linkers were investigated. These included: the NHS-PEG-DBCO linker of 0.65 kDa (Jena Biosciences, Jena, Germany), the 2 kDa NHS-PEG-MeO and 5 kDa NHS-PEG-MeO (both from Iris Biotech GmbH, Marktredwitz, Germany), and the 2 kDa NHS-PEG-DBCO and 5 kDa NHS-PEG-DBCO (both from Nanocs Inc., Boston, MA, USA). Each linker was conjugated onto the nanocarrier surface using three different molecular weight ratios (1:1, 1:10, 1:50) based on the amount of NH_2_. To do this, the number of NH_2_ groups per mg nanocarrier were multiplied by the respective molecular weight of the different linkers and the desired linker ratio. All linker solutions were freshly prepared in DMSO (Merck) for each experiment. After overnight reaction under constant shaking at room temperature, all nanocarrier linker conjugates were washed (3×) with PBS using a strong magnet (neodymium magnet) to remove excess linker species. The final nanocarrier linker products were resuspended in PBS and their concentration was determined by fluorescence calibration. The DBCO-functionalized nanocarriers were conjugated overnight with azide-modified antibodies (see site-directed antibody modification) at room temperature while shaking (in PBS). For this conjugation, the reaction was scaled based on a nanocarrier to azide-antibody ratio of 67:1. For example, 1 mg of nanocarrier was reacted with 14.93 µg of azide-antibody.

**Fluorescence Calibration for Nanocarrier Quantification.** The fluorescence calibration was performed to determine the amount and concentration of all nanocarrier conjugates. Pristine nanocarriers were used as a standard and diluted to a linear fit (100 µg mL^−1^ down to 3.125 µg mL^−1^). All probes were diluted based on the initial mass used. Measurements of the standard and samples were prepared as duplicates in PBS. The Infinite M1000 plate reader (Tecan, Männedorf, Switzerland), with an excitation wavelength of 552 nm and emission of 580 nm, recorded the fluorescence.

**Validation of nanocarrier PEGylation.** The surface PEGylation of the nanocarriers was qualitatively analyzed by two fluorescence-based methods via the plate reader (Infinite M1000, Tecan) and flow cytometry (Attune NxT, Thermo). For this purpose, an Alexa Fluor™ 647 NHS ester (Succinimidylester, Thermo) was used. For the preparation, 100 µg of pristine and PEGylated nanocarriers were reacted with a 50-fold molar excess of the NHS ester (18.75 ng, 1.9 µL of a 10 ng/µL stock) for 1 h at room temperature while shaking. After the incubation, all samples were washed (3×) with PBS using a strong magnet. Pristine nanocarriers incubated with and without the NHS ester were utilized to differentiate between the negative background signal and the positive NHS ester signal, representing the binding towards the primary amines on the nanocarriers. For the plate reader measurement, all samples were resuspended in a final volume of 0.1 mL PBS and 80 µL were pipetted into a black 96-well plate (*n* = 2). The fluorescence was measured with an excitation of 651 nm and an emission of 672 nm. Data sets were calculated from the positive control. For the flow cytometry measurement, 1 µL of nanocarrier sample (~10 µg) was diluted in 1 mL PBS and analyzed by the RL1 channel with an excitation laser of 633 nm and a 670/14 nm band pass filter for emission.

**Site-directed antibody modification.** Antibodies (α-CD11c, clone N418, and α-IgG isotype, clone HTK888, 0.5 mg/mL, both from Biolegend, San Diego, CA, USA) were azide-modified based on the manufacturer’s instructions (Site Click^TM^ Antibody Azido Modification Kit, Thermo). In summary, the concentrated antibody was first reacted overnight with ß-galactosidase at 37 °C to cleave the galactose residues present at the carbohydrate domains of the Fc region. Then the azide groups were attached by incubating the antibody with the GalT (Y289L) enzyme in combination with the UDP-GalNAz donor in an overnight reaction at 30 °C. Finally, the azide-modified antibody (1–2 mg/mL) was purified and concentrated. The modified antibodies were further analyzed for their concentration (Pierce 660 nm Protein Assay), for the validation of their azide-attachment, and for their conjugation onto DBCO-functionalized nanocarriers via copper-free click chemistry.

**Antibody quantification.** The antibody concentrations were specified by using the Pierce 660 nm protein assay according to the manufacturer’s instructions. BSA (bovine serum albumin) was applied as a standard by setting up a dilution series in PBS. The Infinite M1000 plate reader (Tecan) was used to measure the standard and all samples at an optical density of 660 nm, in duplicates.

**Cell line uptake experiments.** For the nanocarrier cell experiments, 1.5 × 10^5^ cells mL^−1^ were cultivated in a 24-well plate in a humidified incubator at 37 °C and 5% CO_2_. After overnight attachment, the cell culture medium was exchanged before each experiment to a fresh medium with or without FBS for 1 to 2 h. This medium exchange was performed so that the cells could adapt to a serum-free environment. All samples were prepared in triplicates and incubated for 2 h with a concentration of 7.5 or 75 µg mL^−1^ in 250 µL of medium per well. The pre-incubation with mouse plasma (collected from 8–12-week-old sexually mature and parasite-free mice, GeneTex, Irvine, CA, USA) was performed for 1 h at 37 °C, shaking in a 1:1 nanocarrier to mouse plasma ratio. Afterwards, the mouse plasma was separated from the samples by using a magnet and 7.5 or 75 µg mL^−1^ in 250 µL medium without FBS were incubated with the cells. After the incubation, 1 mL PBS was used to wash the cells. Then, the cells were detached via 250 µL 2 mM PBS-EDTA for 10 min at 4 °C (DC2.4) or Trypsin-EDTA for 5 min at 37 °C (RAW264.7). Subsequently, 250 µL of medium with FBS were added and the cells were transferred from the well into a 1.5 mL micro tube. The cell viability was determined by using the Zombie Aqua assay (Biolegend, USA). For this assay, the cells were centrifuged at 500× *g* for 5 min and the cell pellet was resuspended in 100 µL Zombie Aqua solution (1:500 diluted in PBS). After an incubation of 15 min at 4 °C in the dark, the Zombie Aqua solution was separated from the cells by centrifugation at 500× *g* for 5 min and the cells were resuspended in 1 mL PBS for analysis using the flow cytometer.

**Flow cytometry.** The measurement of nanocarriers taken up by cells or the detection of covalently bound antibodies on the nanocarrier surface was performed by flow cytometry (Attune™ NxT, Thermo). The nanocarriers were detected by their red fluorescence with the YL1 channel and an excitation laser of 561 nm and a 585/16 nm band pass filter for emission. Viability of the cells was determined by using the Live/Dead fixable Zombie Aqua (Biolegend, USA) with the VL2 channel with an excitation laser of 405 nm and a 512/25 nm band pass filter for emission. The Attune™ NxT Software was applied for data analysis. In a first step, the cell population was selected using an FSC/SSC scatter plot, excluding cell debris. Only the gated events of viable cells were examined by the fluorescence signal expressed as the median fluorescence intensity (MFI), or as the percentage of gated events/cells. For the validation of a correct antibody surface modification of the nanocarriers, a secondary fluorophore-labeled antibody, which was directed against the Fc region of the bound antibody, was chosen. The testing was performed with 2 µg of sample and 1 µg of secondary antibody in 20 µL PBS, and was reacted at 4 °C in the dark for 30 min. The pristine nanocarriers with and without the secondary antibody were applied as adsorption controls. Following incubation, the probes were filled up to 1 mL PBS and analyzed by flow cytometry. After selecting the nanocarrier population by the FSC/SCC scatter plot, the red fluorescence (YL1) was plotted against the SSC. From this selected population, the MFI or the percentage of gated events of the secondary antibody channel were detected by using the BL1 channel with an excitation laser of 488 nm and a 530/30 nm band pass filter for emission.

**Visualization of intracellular localization by cLSM.** Nanocarrier cell interaction was visualized using confocal laser scanning microscopy (cLSM). The experiments were performed on the LSM SP5 STED Leica Laser Scanning Confocal Microscope (Leica, Wetzlar, Germany), composed of an inverse fluorescence microscope DMI 6000CS equipped with a multi-laser combination using a HCX PL APO CS 63 × 1.4 oil objective. The red fluorescence of the nanocarrier samples was excited with the excitation laser 561 nm and detected with an emission filter at 570–599 nm. The cell membrane of the cells was stained with CellMask DeepRed (Thermo) applying the excitation laser 633 nm with a detection at 660–700 nm. For the experimental setup, 5 × 10^4^ RAW264.7 cells in 200 µL DMEM with 10% FBS were seeded in a well of a 15 µ-Slide 8-well glass bottom (ibidi). After overnight attachment in the incubator, the medium was exchanged for 60–90 min, followed by the sample incubation with 75 µg mL^−1^ for 2 h at 37 °C (DMEM medium with FBS, without FBS, and pre-incubated with mouse plasma and resuspended in medium without FBS). After the incubation, the cells were washed once with PBS and the cell membrane was stained for 5 min in the dark (1:1.000 in PBS) before live cell imaging. The image acquisition was performed by using ImageJ (v1.52n).

**Incubation and Visualization of Mouse Plasma.** The murine biomolecular corona proteins of the nanocarriers were analyzed by SDS-PAGE and silver staining (both from Thermo). For this analysis, the samples were incubated with the mouse plasma at a 1:1 volume ratio for 1 h at 37 °C while shaking. Subsequently, the mouse plasma was removed and the samples were washed (3×) with PBS by using a magnet. Adsorbed mouse plasma proteins were detached from the samples by resuspending the nanocarrier pellet in 100 µL desorption buffer (2% (*w*/*v*) SDS + 62.5 mM Tris-HCl in 3 mL deionized water) for 10 min at 70 °C while shaking. Desorbed proteins were recovered from the nanocarrier samples by applying a magnet. The total amount of denatured proteins in the supernatant was quantified by Pierce 660 nm protein assay and 2 µg were used for SDS-PAGE, visualized by silver staining.

**Multi-angle dynamic light scattering**. For the detection of the size (diameter in nm) and size distribution (polydispersity index, PDI), all nanocarriers were analyzed by multi-angle dynamic light scattering. The measurements were performed on an ALV spectrometer consisting of a goniometer and an ALV-5004 multiple-tau full-digital correlator (320 channels) at 20 °C and 7 (dynamic) angles ranging from 30° to 150°. The light source was a He-Ne Laser (wavelength of 632.8 nm). The samples were prepared by diluting 1 µL of unfiltered dispersion with 1 mL PBS, which was previously filtered through membrane filters (GS Millipore) with a pore size of 2.0 µm.

**Zeta potential.** The surface charge of the nanocarriers was detected using a Zeta Sizer Nano Series (Malvern Instruments GmbH, Herrenberg, Germany). All samples (10 µL) were diluted in a 1 mM potassium chloride solution (1 mL) and measured in technical triplicates.

**TEM.** The FEI Tecnai F20 transmission electron microscope (operating at a voltage of 200 kV) was used to investigate the morphology of the nanocarriers. To achieve this, all samples were diluted to a particle concentration of 75 µg mL^−1^ in IMDM medium containing 5% FBS, and 4 µL were pipetted on a carbon-coated grid. The excess solvent was blotted away with filter paper.

**In solution digestion.** SDS was removed from the protein corona samples by using Pierce^TM^ Detergent Removal Spin Columns (Thermo Fisher Scientific, Waltham, MA, USA) according to the manufacturer’s instructions. Next, protein samples were subjected to an in-solution tryptic digest as a preparation for the LC-MS proteomic measurements. The digestion protocol was carried out as described previously with modifications [30,31]. Briefly, 10 to 25 µg of protein were precipitated and washed twice by using the ProteoExtract Protein Precipitation Kit (CalBioChem, Darmstadt, Germany) according to the manufacturer’s instructions. After letting the protein pellet dry for 5 to 10 min, the protein was solubilized in 0.1% RapiGest SF surfactant (Waters Corporation, Germany, dissolved in 50 mM ammonium carbonate buffer), at 80 °C for 15 min. Disulfide bonds were reduced with a 5 mM final concentration of dithiothreitol (Sigma, Roedermark, Germany) at 56 °C for 45 min. Thiol alkylation was performed with a 500 mM final concentration of iodoacetamide (Sigma, Germany) at room temperature for 1 h in the dark. Subsequently, the digestion was carried out with trypsin in a mass ratio of 50:1 (protein:trypsin) at 37 °C for a maximum of 18 h. The digestion was stopped by lowering the pH of the peptide solution with 2 µL of hydrochloric acid (Sigma, Germany). To remove aggregates, the samples were centrifuged at 13,000× *g* for 15 min at 4 °C and the supernatant was transferred to new tubes for measurement.

**Liquid chromatography-electrospray ionization mass spectrometry (LC-MS).** Peptide samples were spiked with 50 fmol μL^−1^ HI3 *E. coli* Standard (Waters Corporation, Germany), and the total volume was adjusted with LC-MS grade water (Merck, Darmstadt, Germany). The absolute quantification performed in these measurements was based on a previously published protocol [32]. The LC-MS measurements were carried out as previously described in the work of our group [16,31]. A nanoACQUITY UPLC system coupled to a Synapt G2Si mass spectrometer (both Waters Corporation, Germany) was utilized, operating the measurements with MassLynx 4.1 (Waters Corporation). The UPLC was equipped with a C18 nanoACQUITY trap column (5 μm, 180 μm × 20 mm) and a C18 analytical reversed-phase column (1.7 μm, 75 μm × 150 mm; both Waters Corporation, Germany). The samples were injected at a flow rate of 0.3 μL min^−1^ and separated by a gradient of 2% to 37% of mobile phase B over 70 min. The solvents for the gradient consisted of (A) 0.1% (*v*/*v*) formic acid in LC-MS grade water and (B) 0.1% (*v/v*) formic acid in acetonitrile (Biosolve, Neuss, Germany). The reference components Glu-Fibrinopeptide and LeuEnkephalin (both Sigma, Germany) were injected at a flow rate of 0.5 μL min^−1^. The mass spectrometer was equipped with a NanoLockSpray for source by electrospray ionization (ESI) and configured for the positive mode. The measurements were conducted in the resolution mode with data-independent acquisition (MS^E^) in a mass to charge range of 50–2000 Da, scan time of 1 s, ramped trap collision energy from 20 to 40 V, and data acquisition of 90 min. All samples were measured in technical triplicates.

**Protein identification.** Protein identification was performed by using the proteomics analysis software Progenesis QI 2.0 (Nonlinear Dynamics). The procedure was described previously [16]. Following settings were applied to process the data: Noise reduction threshold for low energy defined as 120 counts, elevated energy as 25 counts, and peptide intensity as 750 counts. The utilized protein database consisted of the murine proteome with reviewed proteins, which were downloaded from uniport (swiss prot). The sequence information for the Hi3 *E. coli* standard for absolute quantification was added to the protein database file. The following settings were used to identify the proteins from the peptides: one missed cleavage, maximum protein mass of 600 kDa, fixed carbamidomethyl modification for cysteine, variable oxidation for methionine, a minimum of three assigned fragments per peptide, a minimum of two assigned peptides per protein, a minimum of five assigned fragments per protein, and a score parameter below 4. The protein quantification in fmol was calculated based on the TOP3/Hi3 approach [33]. A table with all identified proteins is provided in the Supplementary Information section.

**Generation of bone marrow-derived dendritic cells.** Bone marrow-derived dendritic cells (BMDC) were differentiated from progenitor cells (BM cells) isolated from the bone marrow of 8- to 12-week-old C57BL/6J mice as previously described [34]. Briefly, bone marrow was obtained by flushing the femur and tibia with cell culture medium (Iscove’s Modified Dulbecco’s Medium (IMDM) containing 5% FBS (PAN), 2 mM GlutaMax (Thermo Fisher Scientific), 100 U mL^−1^ penicillin, 100 µg mL^−1^ streptomycin, and 50 µM β-mercaptoethanol (Sigma-Aldrich) through a 26-gauge needle. BM cells were washed with cell culture medium and red blood cells were lysed using Gey’s lysis buffer (155 mM NH_4_Cl, 10 mM KHCO_3_, 100 µM EDTA) for 1 min at 4 °C. BM cells were washed again and seeded in non-TC-treated 10 cm-petri dishes (2 × 10^6^ in 10 mL per dish). Fifty percent of cell culture medium was replenished on days 3 and 6. Nonadherent and loosely adherent immature BMDCs were harvested on day 7 and used for uptake experiments.

**BMDC uptake experiments.** Harvested BMDCs were washed with FBS-free cell culture medium and reseeded in 24-well suspension culture plates (10^5^ per well) in 500 µL cell culture medium, either supplemented with 5% FBS or with 5% mouse plasma. Subsequently, cells were incubated with 3 µg mL^−1^ of different nanocarrier formulations for 20 h at 37 °C and 7.5% CO_2_, harvested, and analyzed by flow cytometry.

**Conventional DC type 1 uptake experiment.** Spleen cells were prepared as previously described [35]. Briefly, spleens of C57BL/6J mice were dissected and mechanically ground through a 40 µm cell strainer. Red blood cells were subsequently lysed using a hypotonic buffer (155 mM NH_4_Cl, 10 nM KHCO_3_, 100 μM EDTA-disodium, pH 7.4) for 30 s and washed using cell culture medium (Iscove’s Modified Dulbecco’s Medium (IMDM) supplemented with 5% fetal bovine serum (FBS), 1% penicillin (100 U mL^−1^), and streptomycin (100 µg mL^−1^). Isolated spleen cells were subsequently seeded in 48-well plates (10^6^ per well) in 500 µL cell culture medium without FBS supplemented with 5% mouse plasma. Different nanocarrier formulations were added (3 µg mL^−1^) and cells were incubated for 20 h at 37 °C and 7.5% CO_2_. Spleen cells were harvested and washed (1% FBS in PBS). Fc receptors were blocked with anti-CD16/CD32 (clone 2.4G2) for 10 min. To differentiate the cell types via flow cytometry, spleen cells were incubated with fluorophore-labelled cell type-specific antibodies for 30 min: anti-CD11c (clone N418), anti-CD11b (clone M1/70), anti-CD172a (clone P84), anti-CD8α (clone 53–6.7), anti-I-A/I-E (MHCII, clone M5/114.15.2), anti-Siglec-H (clone 551), anti-CD19 (clone 6D5), anti-CD3ε (clone 145–2C11), anti-CD14 (clone Sa14-2), anti-NK1.1 (clone PK136), and anti-Ly-6G (clone 1A8). Flow cytometric analysis was performed using the Attune NxT (Thermo Fisher Scientific) and analyzed with FlowJo software v10.7.1. Conventional DCs type 1 were defined as CD19^−^ CD3^−^ CD14^−^ NK1.1^−^ Ly-6G^−^ MHCII^+^ CD11c^+^ CD11b^−^ CD172a^−^ CD8α^+^.

**Statistical analysis.** For the PEGylated nanocarrier uptake in the cell lines, the pristine nanocarriers of each medium condition (medium^-FBS^, medium^+FBS^, pre-incubated in mouse plasma) were statistically compared to the PEGylated samples from the corresponding medium condition using GraphPad Prism 9. One-way analysis of variance (ANOVA) was followed by a Dunnett’s multiple comparison test with a confidence interval as indicated in each figure. The CD11c/isotype fold changes were statistically compared to each other using the Brown–Forsythe and Welch ANOVA Test.

## 3. Results

The experimental workflow can be outlined as follows: we first investigated the stealth effect by coating our magnetic and red-fluorescent nanocarriers (magnetic hydroxyethyl starch particles or mgHES) with different PEG ratios. These PEGylated nanocarriers were then examined in cell culture with macrophages and DCs. Based on these findings, the PEG-induced stealth effect was then combined with the DC targeting ability of antibodies and termed *stealth targeting*. Both, the stealth effect and stealth targeting nanocarriers were analyzed based on their physicochemical characteristics (ζ-potential, MADLS, and TEM), their interaction with macrophages and DCs (flow cytometry and cLSM), and their proteomic biomolecular corona composition (SDS-PAGE and LC-MS).

Initially, two different PEG linkers (NHS-PEG_x_-MeO) of 2 and 5 kDa molecular weight were covalently immobilized onto the surface of the amine-functionalized nanocarriers. Three PEG excess ratios were then examined in order to achieve a sufficient stealth effect. This stealth effect, namely a reduced uptake of the PEGylated samples compared to the pristine nanocarriers, was investigated in highly phagocytic cells (macrophage cell line, RAW264.7), as well as in the cells of interest (DC line, DC2.4) and further analyzed by flow cytometry. In addition, primary DCs, such as bone marrow-derived DCs (BMDCs) and conventional DCs type 1 (cDC1) from the spleen, were analyzed for their potential to take up antibody-functionalized nanocarriers.

**Figure 1:**
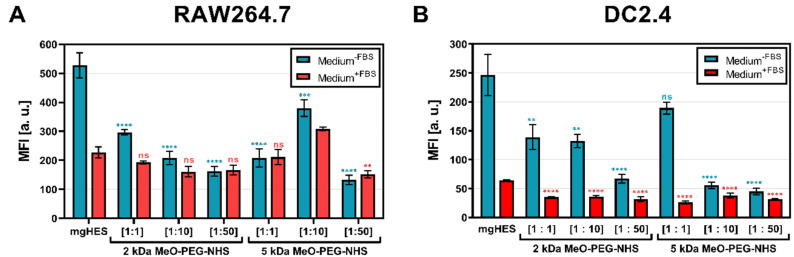
**Stealth effect of PEGylated nanocarriers in macrophages and dendritic cells.** The cell uptake study in (**A**) RAW264.7 macrophages or (**B**) DC2.4 dendritic cells was conducted using a sample concentration of 7.5 µg mL^−1^ and incubation time of 2 h at 37 °C. Autofluorescence is subtracted from each data set. Only viable cells are gated. Values are given as mean ± SD (*n* = 3). Each condition was compared to the respective control (pristine mgHES) and significance was given with *p* < 0.05 using a one-way ANOVA followed by a Dunnett’s multiple comparison test. ns = not significant, *p* < 0.01 **, *p* < 0.001 ***, *p* < 0.0001 ****.

First, three molar ratios of the linkers were evaluated (Figure 1A,B). Furthermore, the use of culture medium with and without fetal bovine serum (FBS) highlighted the role of additional proteins that can form a biomolecular corona on the nanocarrier surface, potentially influencing the nanocarrier cell interaction [36]. Each PEG-modified (PEGylated) nanocarrier formulation was taken up to a lower rate by both cell lines when incubated in medium without FBS (blue bars). Given this medium condition, the stealth effect was found to be more pronounced for both cell lines at higher PEG ratios than at lower ones. In the medium supplemented with additional FBS proteins (red bars), the overall uptake of the samples was reduced compared to the medium without FBS. Here, only slightly lower values, or slightly increased values (1:10 ratio of the 5 kDa sample in RAW cells, Figure 1A) were detected. A similar finding could be observed when analyzing the frequency of NC^+^ cells (Supporting Information Figure S1A,B).

In this case, the nanocarrier PEGylation was limited to the number of surface-NH_2_ sites (3 nmol mg^−1^) accessible for conjugation. Nevertheless, the cell uptake studies in both cell lines revealed that higher PEG ratios with sufficient reaction rates of NHS to NH_2_ were better able to convey the stealth effect. Therefore, higher PEG to NH_2_ ratios were able to fully saturate the NHS reaction on the nanocarrier surface. The difference in the PEGylation ratios could be demonstrated by using an Alexa Fluor^TM^ 647 NHS-ester. For this purpose, the difference in the fluorescence labeling between PEGylated and pristine nanocarriers was evaluated (see Material and Methods section for a detailed description). Following the conjugation, the Alexa Fluor^TM^ 647-based nanocarrier fluorescence was measured by flow cytometry and the plate reader (Figure S2A,B). A decreasing fluorescence signal was observed for increasing PEG ratios (10× and 50×) indicating that less primary amines were available to react with the Alexa Fluor^TM^ 647 NHS-ester compared to the equimolar ratio (1:1). Given the 3 nmol NH_2_ per mg nanocarrier and assuming a sphere shape, in theory, the mgHES nanocarriers are equipped with 0.4 NH_2_ target sites per nm^2^. Therefore, at a full conversion of amines to NHS linker reactions, 0.4 PEG chains per nm^2^ can be assumed. In summary, the best stealth properties could be achieved when PEGylation was performed at the molar ratio of NH_2_ on nanocarriers to PEG linker ratio of 1:50. Li et al. showed that a PEG grafting density of 0.2 to 2.5 chains nm^−2^ on polymeric ovalbumin nanocapsules with 2 kDa PEG gave rise to the formation of an intermediate mushroom and to a dense brush conformation [37]. The ovalbumin nanocapsules evaluated in the aforementioned study, that were modified with 5 kDa PEG, acquired the brush conformation even at a low density of 0.2 chains nm^−2^ due to the higher molecular weight. In comparison to the ovalbumin nanocapsules, the PEGylated nanocarriers analyzed here also seem to differ in their PEG conformation, with a transition from the less-favorable mushroom conformation to the desired brush conformation, resulting in the greatest stealth effect for a complete amine to PEG conversion in macrophages and DCs. Previous PEGylation studies showed that the PEG-induced stealth effect is highly dependent on the density and conformation of the surface PEG chains. These parameters affect the formation of a PEG barrier on the nanocarrier surface, and therefore decrease opsonization and increase circulation time [38,39]. While the mushroom conformation with a larger PEG chain motion and unoccupied spaces on the surface is more susceptible to opsonins, the brush conformation with a limited PEG mobility and an increased surface coverage has been shown to reduce the interaction between the nanocarrier and the opsonins [14,40].

In addition, the shortest PEG linker (0.65 kDa) applied in our previous studies [29], was tested in terms of RAW264.7 cell uptake (Figure S1C,D). In comparison to the 2 and 5 kDa PEG linkers (Figure 1), the 0.65 kDa PEG linker showed higher uptake values (both for the MFI and frequency of NC^+^ cells) when FBS was present in the medium. This less pronounced stealth effect, compared to the 2 and 5 kDa PEGylated nanocarriers (50x excess), could also be influenced by the difference in the reactive group pointing to the cells. Namely, the methoxy (MeO) or the dibenzocyclooctyne (DBCO) groups, of which the latter one is necessary for clicking an azide-functionalized antibody onto the nanocarrier’s surface.

**Figure 2:**
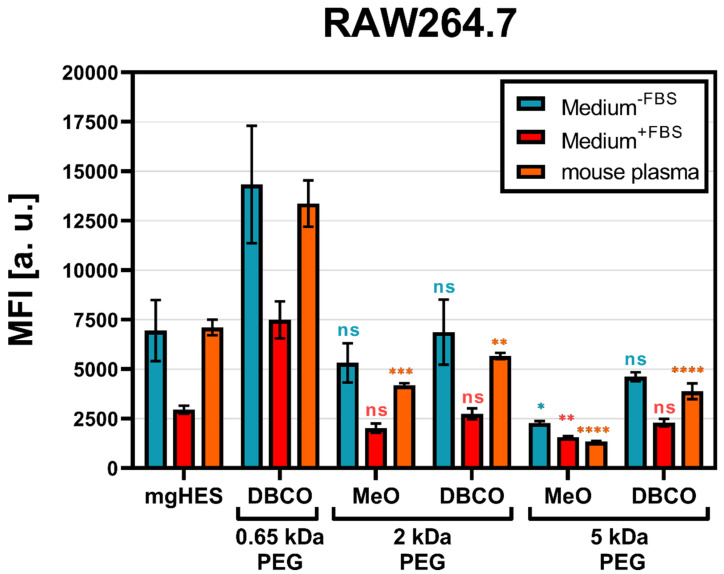
**Longer PEG chains reduce nanocarrier uptake by RAW264.7 cells**. Nanocarriers were incubated with RAW264.7 cells in either a medium with or without FBS, or pre-incubated with mouse plasma with a sample concentration of 75 µg mL^−1^ and incubated for 2 h at 37 °C. Autofluorescence was subtracted from each data set. Only viable cells were analyzed. Values are given as mean ± SD (*n* = 3). Each PEGylated sample was compared to the pristine nanocarrier group (mgHES) and significance was given with *p* < 0.05 using a one-way ANOVA followed by a Dunnett’s multiple comparison test. ns = not significant, *p* < 0.05 *, *p* < 0.01 **, *p* < 0.001 ***, *p* < 0.0001 ****.

With that in mind and according to the findings described above, we decided to further investigate the 50-fold molar excess ratio of the different PEGylated nanocarrier formulations with either the MeO (for the 2 and 5 kDa linkers), or the DBCO-reactive end group (for the 0.65, 2, and 5 kDa linkers) with respect to RAW264.7 cell uptake. Additionally, the PEGylated samples were pre-incubated with mouse plasma for 1 h at 37 °C in order to mimic a more physiological condition (Figure 2).

As a result, we found that the PEGylated samples in medium without FBS and pre-incubated in mouse plasma showed almost no differences, indicating that the murine biomolecular corona is hardly involved in conveying or preventing the stealth effect. Overall, the uptake analysis showed that in every sample group the frequency of NC^+^ cells was above 80% (Figure S3). However, based on the MFI, we observed that the PEGylated samples were taken up to a much lower degree. For both, the medium without FBS and the mouse plasma pre-incubation, the greatest stealth effect could be observed for the higher molecular weight PEG linker (5 kDa) with the MeO end group. Carrying DBCO at the end group resulted in a generally higher uptake compared to MeO. This influence of the DBCO group was particularly evident for the shortest PEG linker. The FBS-containing medium showed weaker but comparable results to the other two medium conditions. However, the use of bovine-derived serum in a murine cell uptake study is relatively artificial, since it does not resemble a physiological environment and can bias the cell uptake results [41].

**Figure 3:**
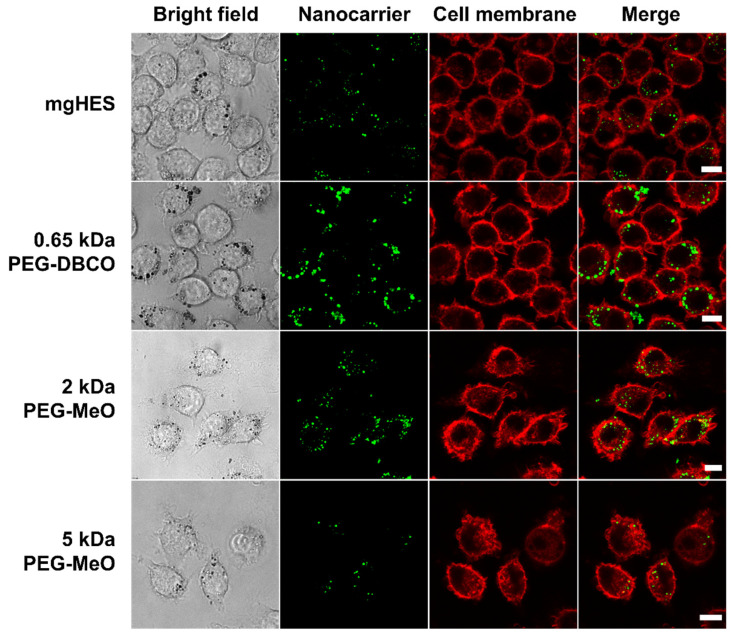
**Longer PEG chains reduce internalization by macrophages.** Mouse plasma pre-incubated nanocarriers were incubated with 5 × 10^4^ RAW264.7 cells for 2 h at 37 °C with a sample concentration of 75 µg mL^−1^ in DMEM medium without FBS. Live cell images are shown. All scale bars represent 10 µm.

Confocal laser scanning microscopy was used to further depict the stealth effect and the cellular localization of our nanocarriers that were either sticking to the cell membrane or being internalized (Figure 3). Therefore, the uptake of pristine and PEGylated nanocarriers, either pre-incubated in mouse plasma (no FBS present in medium), or in medium with 10% FBS was investigated in RAW264.7 cells (Figure S4). Confocal laser scanning microscopy imaging on dendritic cells (DC2.4) was performed in a previous study showing preferential uptake of anti-CD11c-functionalized mgHES nanocarriers [29].

Visually, almost all nanocarrier formulations were internalized by the cells with only a few localized at the cell membrane. Only the 5 kDa PEGylated sample could be clearly distinguished from the other sample groups. Here, we found a lower number of nanocarriers as well as a weaker fluorescence signal. In comparison to the uptake in FBS-containing medium, a generally lower nanocarrier uptake of all samples could be observed. Here, the longer PEG chains with 2 and 5 kDa led to a reduction in cell internalization. The stealth properties of the PEG linkers with the DBCO-reactive group could not be visualized microscopically, and were therefore not included in the 2 and 5 kDa linkers.

**Figure 4:**
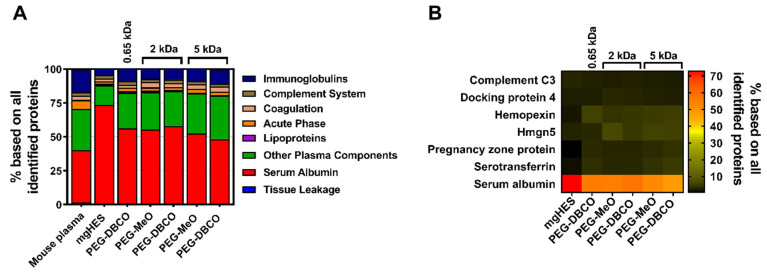
**Nanocarrier PEGylation shows little influence on the adsorption profile of the biomolecular protein corona**. Adsorbed corona proteins were analyzed by LC-MS and either sorted into eight protein groups (**A**), or individually plotted as a heatmap (**B**). Hmgn5 = High mobility group nucleosome-binding domain-containing protein 5.

In a next step, the influence of the biomolecular corona on the stealth effect was determined by quantitatively analyzing the murine proteome of pre-incubated, PEGylated nanocarriers (all with a 50-fold molar excess of linker) via label-free mass spectrometry (LC-MS). All identified proteins could be correlated to eight protein groups and the respective values were plotted as the percentage based on all identified proteins (Figure 4A). In comparison to the composition of the mouse plasma control, the percentage of serum albumin within the protein corona of the pristine nanocarriers (mgHES) increased significantly while the frequency of proteins of all other groups slightly decreased. A similar finding could be observed for all PEGylated samples irrespective of the PEG linker length used, even though the elevation in serum albumin content was not as pronounced as for the pristine nanocarriers. Qualitative analysis using SDS-PAGE further revealed serum albumin to be the strongest band with a molecular weight of about 62 kDa as detected by silver staining (Figure S5A).

Additionally, the top seven most abundant corona proteins detected in each group were plotted as a heatmap (Figure 4B). In parallel to the protein group analysis, serum albumin was found to be by far the most abundant corona protein for every nanocarrier formulation. While serum albumin presence was detected in the range of 48% (5 kDa PEG-DBCO) to 72% (mgHES), the other six most abundant proteins were observed with a relative abundance of less than 7% each. The whole mouse plasma control group showed a comparable distribution with the most abundant proteins (Figure S6).

Apart from complement C3, none of the other abundant murine proteins is thought to influence uptake by macrophages. C3 coated on nanocarriers, however, can lead to an increased internalization of NCs by macrophages through complement receptor ligation when being activated by pathway-specific C3 convertases [42,43]. However, the relatively low abundance of C3 on each nanocarrier formulation (~2%) indicates that C3-induced uptake by macrophages only plays a minor role in this carrier system. Walkey et al. demonstrated that higher PEG grafting densities control a decrease and change in the composition of the protein corona, where the nanoparticle size and the PEG density together determine the macrophage uptake [44]. In contrast, PEGylation of the nanocarrier formulations described here increased the rate of protein adsorption in comparison to the pristine nanocarriers (Figure S7). Nevertheless, this increase in corona protein adsorption did not diminish the stealth effect of PEGylated nanocarriers. In addition, the physicochemical properties of the PEGylation with and without a protein corona were evaluated with respect to nanocarrier size and surface charge (Table S1). Multi-angle dynamic light scattering revealed a larger size of pristine nanocarriers compared to the PEGylated formulations in protein-free media, and a smaller particle size under protein corona conditions. This might be due to changes in the light scattering properties and/or a reduction in aggregation after PEGylation. Regarding the surface charge of the nanocarriers, all samples presented a rather neutral zeta potential, which consistently appeared slightly negative for all mouse plasma pre-incubated nanocarrier formulations. In addition, TEM was used to visually confirm the nanocarrier size and morphology (Figure S8). There were no visual differences between the pristine and PEGylated nanocarriers detected. All formulations appeared to form small cluster duplets with a diameter of about 200 nm. In summary, the experiments conducted during this study exhibited a reduced but not fully diminished protein corona formation following PEGylation of mgHES nanocarriers, which in turn did not have an influence on the PEG-induced stealth properties.

In addition to avoiding non-specific clearance of nanocarriers by phagocytes, conjugating active targeting moieties onto nanocarriers is the main prerequisite for a precise targeting of distinct DC subsets. Each DC subset is characterized by its ability to migrate, cytokine secretion, and antigen presentation. Hence, the selection of a distinct subtype critically affects a realistic DC vaccination approach. Applying the pan-DC marker CD11c as a target receptor has already underlined the role of DCs in enhancing humoral responses [45,46,47,48]. Prior to the investigation of primary DCs, the influence of the length of the different DBCO-containing PEG linkers on the antibody-based targeting ability of the nanocarrier formulations used here were investigated in the DC2.4 cell line. In order to evaluate the stealth targeting, azide-modified CD11c antibodies were covalently bound onto the DBCO-functionalized surface as described previously [29]. Additionally, the corresponding isotype control antibody was included as a control for non-specific, Fc-mediated uptake. This control antibody was conjugated to each NC-PEG_X_-DBCO linker group to differentiate between specific Fab- and undesired Fc-mediated cell binding. Conjugates with the 0.65 kDa PEG-DBCO linker were generated using a 10-fold molar excess of the linker, while the 2 kDa and 5 kDa molecular weight NC-PEG_X_-DBCO linkers were conjugated with a 50-fold molar excess. Azide-modified antibodies were conjugated at a nanocarrier to antibody weight ratio of 67:1.

**Figure 5:**
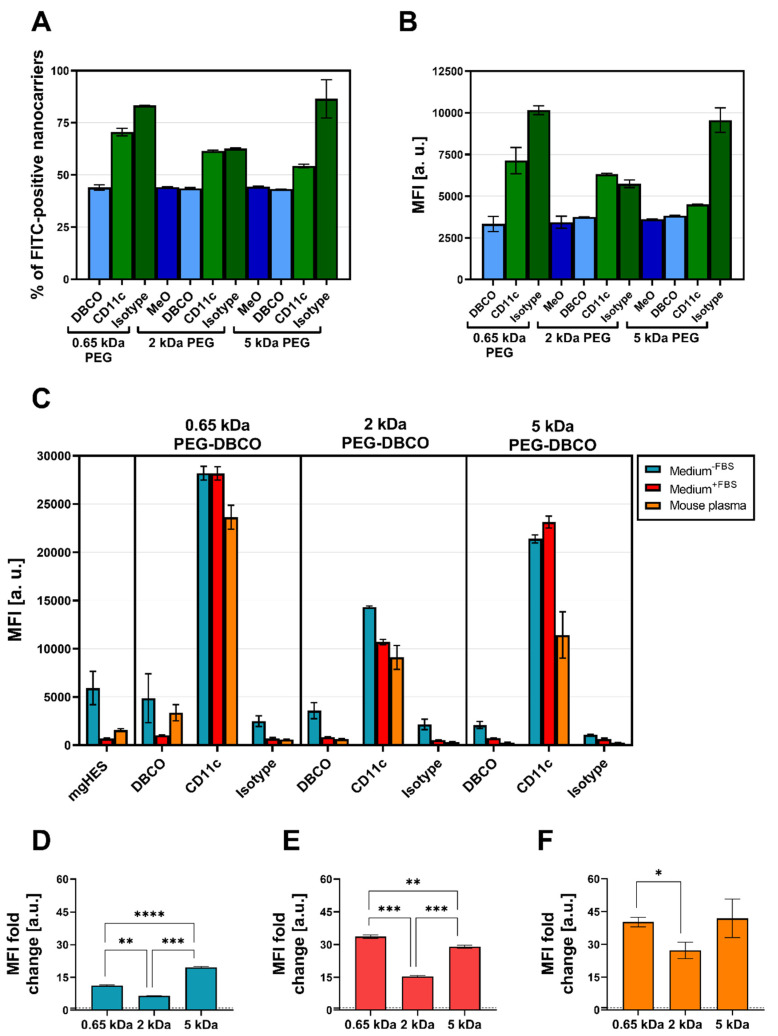
**The PEG length affects antibody-based targeting in DC2.4 cells.** Secondary antibody testing by flow cytometry qualitatively validated the presence of covalently bound antibodies on the nanocarrier surface, presented here as the percentage of positive events (**A**), and as the median fluorescence intensity [arbitrary units] (MFI [a. u.]) (**B**). Values are given as mean ± SD (*n* = 2). DC2.4 cells were incubated with nanocarriers (75 µg mL^−1^) functionalized with antibodies and PEG linkers of varying lengths (0.65 kDa, 2 kDa, 5 kDa) for 2 h in culture medium without FBS, with 5% FBS, or pre-incubated with mouse plasma, and the uptake was quantified by analyzing the MFI (**C**). Values are given as mean ± SD (*n* = 3). For each cell culture medium condition, the MFI values obtained by analyzing anti-CD11c-functionalized nanocarriers were normalized to their isotype controls and different PEG linker lengths were compared (without FBS (**D**), with FBS (**E**), pre-incubated in mouse plasma (**F**)). Significance was given with *p* < 0.05 using a Brown–Forsythe and Welch ANOVA test), *p* < 0.05 *, *p* < 0.01 **, *p* < 0.001 ***, *p* < 0.0001 ****.

Flow cytometry was used to validate the successful covalent binding of the CD11c and isotype antibodies on the PEG-DBCO linker-modified nanocarrier surface (Figure 5A,B). To achieve this, antibodies immobilized on the nanocarrier surface were stained with an anti-hamster FITC secondary antibody. The binding of the secondary antibody, either via adsorption onto the nanocarrier’s surface (no covalent-bound antibody present), or a positive, non-covalent binding towards the Fc region of the covalently attached antibodies, enabled a qualitative detection method. In this way, we confirmed the presence of the covalently bound antibodies on the surface for each formulation. Subsequently, the CD11c targeting ability of functionalized nanocarriers was examined in terms of uptake by DC2.4 cells under three different medium conditions via flow cytometry (Figure 5C). For each condition, the targeting ability of anti-CD11c-modified nanocarriers was clearly distinguishable from the isotype control-functionalized formulations. Only the CD11c nanocarrier conjugates showed a significant uptake by the DC2.4 cells, while the pristine nanocarriers, the corresponding DBCO linker conjugates, and the isotype conjugates remained at lower values. Comparing the three CD11c nanocarrier conjugate groups, the highest uptake was observed for the 0.65 kDa PEG-CD11c, followed by the 5 kDa PEG-CD11c, and 2 kDa PEG-CD11c being the weakest for culture medium with or without FBS. Applying mouse plasma pre-incubated nanocarriers, only a minor difference between the 2 and 5 kDa PEG-CD11c could be observed, while the 0.65 kDa PEG-CD11c exhibited a substantial uptake. The anti-CD11c/isotype control ratio based on the respective MFI values was calculated in order to evaluate the influence of different PEG chain lengths on the specificity of antibody-mediated targeting (Figure 5D–F). Among the three medium conditions, the ratio demonstrated a particularly low influence of the Fc region when applying the 5 kDa CD11c-functionalized nanocarriers, which was even more pronounced under medium conditions that led to protein corona formation (Medium^+FBS^ E, or mouse plasma pre-incubated, F).

**Figure 6:**
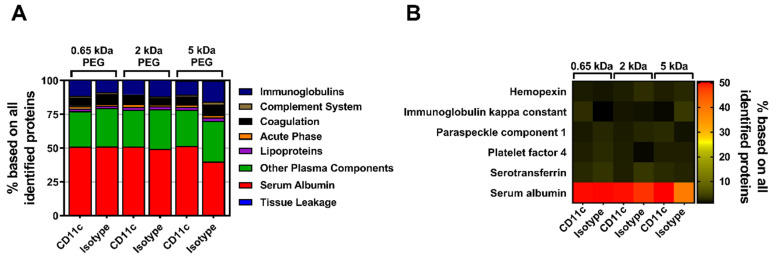
**Antibody surface-modifications have no influence on the murine biomolecular protein corona composition.** Adsorbed corona proteins were analyzed by LC-MS and either sorted into eight protein groups (**A**), or individually plotted as a heatmap (**B**).

In parallel to the MeO-PEG-coupled nanocarriers, the influence of the biomolecular corona of anti-CD11c-functionalized nanocarriers on their targeting ability was analyzed and quantitative LC-MS was performed. Again, all identified proteins were assigned to eight protein groups and the respective values were plotted as the percentage based on all identified proteins (Figure 6A). No major differences between the three molecular weight PEG groups with either the CD11c targeting or the isotype control antibody could be detected. All of the individual groups exhibited serum albumin, which had been highly adsorbed onto the nanocarrier’s surface.

Likewise, the qualitative SDS-PAGE detection of desorbed corona proteins showed serum albumin as the most abundant protein as visualized by silver staining (Figure S5B). Moreover, by depicting the top six corona proteins we further highlighted that serum albumin has the highest abundance of all identified proteins (Figure 6B). As seen before with PEGylation, the analysis of the murine biomolecular corona showed that the surface-modification of the nanocarriers with PEG, or in combination with antibodies only, slightly influenced the protein adsorption profile. In general, only a low number of proteins adsorbed onto the nanocarrier’s surface with very little influence on the stealth targeting ability.

**Figure 7:**
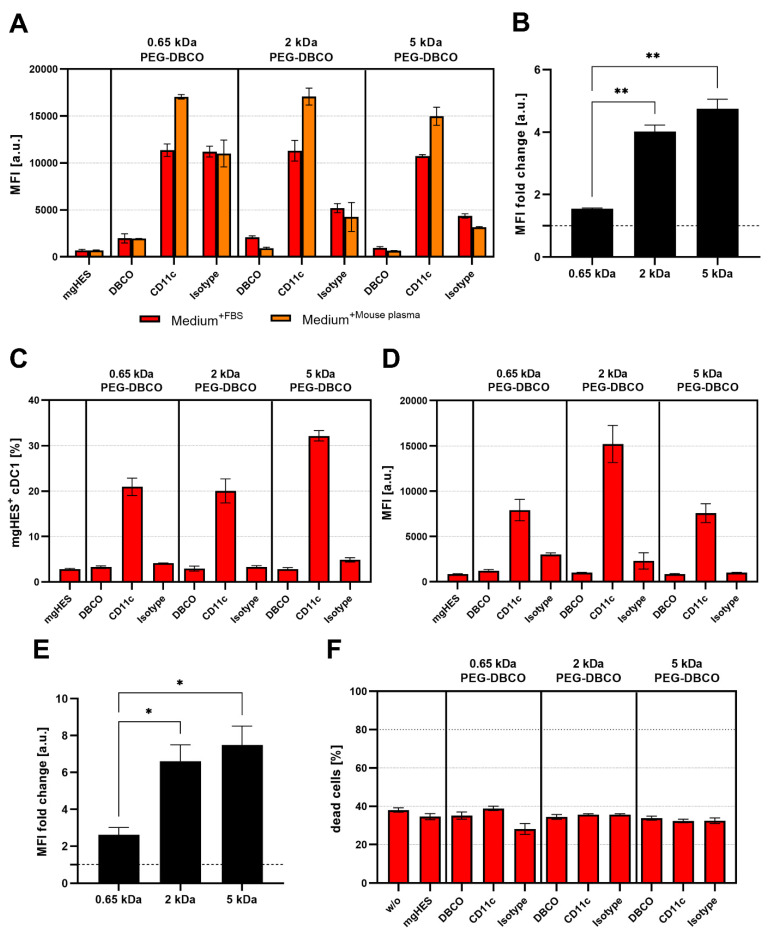
**The PEG length affects antibody-based targeting in primary dendritic cells.** (**A**) BMDCs were incubated with nanocarriers (3 µg mL^−1^) functionalized with antibodies and PEG linkers of varying lengths (0.65 kDa, 2 kDa, 5 kDa) for 20 h in culture medium containing either 5% FBS or 5% mouse plasma, and uptake was quantified by analyzing the median fluorescence intensity (MFI) by flow cytometry. (**B**) MFI values obtained by analyzing anti-CD11c-functionalized nanocarriers cultured in medium + 5% mouse plasma were normalized to their isotype controls and different PEG linker lengths were compared. Data represent mean ± SD. Significance was given with *p* < 0.05 (Brown–Forsythe and Welch ANOVA test), *p* < 0.01 **. (**C**,**D**) Splenocytes were incubated with nanocarriers (3 µgmL^−1^) functionalized with antibodies and PEG linkers of varying lengths (0.65 kDa, 2 kDa, 5 kDa) for 20 h, and uptake by conventional DCs type 1 (cDC1) was quantified by analyzing the percentage of NC^+^ cells (**C**), and MFI (**D**), by flow cytometry. (**E**) MFI values obtained by analyzing anti-CD11c-functionalized nanocarriers were normalized to their isotype controls and different PEG linker lengths were compared. Data represent mean ± SD. Significance was given with *p* < 0.05 (Brown–Forsythe and Welch ANOVA test), *p* < 0.05 *. (**F**) Toxicity of nanocarrier formulations was documented by staining of splenocytes with LIVE/DEAD Fixable Aqua Dead Cell Stain after 20 h of co-incubation and subsequent flow cytometric analyses.

While conducting the uptake studies using the DC2.4 cell line, we also analyzed the influence of the PEG linker length on the uptake of anti-CD11c-functionalized nanocarriers in primary DCs, such as BMDCs and cDC1s of the spleen (Figure 7A–E). In contrast to the uptake studies in DC2.4 cells, the targeting ability of anti-CD11c-functionalized nanocarriers was reduced when incubated with BMDCs (Figure 7A). This was even more pronounced for incubation in the presence of FBS. However, the targeting ability, in terms of the anti-CD11c/isotype control ratio, was significantly increased for the longer PEG-DBCO linkers 2 kDa and 5 kDa by 4.0-fold and 4.75-fold, respectively, compared to 0.65 kDa PEG-DBCO (1.55-fold) (Figure 7B). When analyzing cDC1s within whole splenocytes cultures, anti-CD11c-functionalized nanocarriers exhibited a substantial targeting ability compared to pristine, DBCO-, or isotype-functionalized nanocarriers (Figure 7C–E). The uptake of IgG-functionalized nanocarriers was almost reduced to background levels in contrast to BMDC cultures, in terms of frequency of NC^+^ cells (Figure 7C), as well as MFI (Figure 7D). In line with the data obtained from the incubation with DC2.4 cells and BMDCs, the PEG-DBCO linker length significantly influenced the anti-CD11c-mediated uptake of nanocarriers. While the 0.65 kDa linker only led to a 2.6-fold higher uptake rate compared to the isotype control, 2 kDa and 5 kDa resulted in significantly higher specific uptake by 6.6-fold and 7.5-fold, respectively (Figure 7E). Additionally, the toxicity of different nanocarrier formulations was evaluated by co-incubation with splenocytes for 20 h and staining of dead cells with LIVE/DEAD Aqua Dead Cell Stain (Figure 7F). Flow cytometric analyses revealed no significant changes in the percentage of dead cells when comparing all nanocarrier formulations under study.

## 4. Conclusions

In this study, we have furthered our research on our previously established antibody–nanocarrier conjugation strategy by comparing different molecular weight PEG linkers for the spacing between the antibody and the nanocarrier surface. This spacing determines the flexibility of the attached antibody and directly influences the targeting ability of the nanocarrier. Accordingly, our findings systematically highlight the differences between a DC cell line and primary DC subsets regarding the influence of PEG linker lengths upon the antibody-based targeting ability of nanocarriers. In particular, CD11c-based DC2.4 targeting proved to be most efficient when using the shortest linker (0.65 kDa PEG), whereas a distinct subset-specific cDC1 targeting in primary cells requires a longer linker (5 kDa PEG). This discrepancy probably explains the misinterpretation of in vitro data generated using DC cell lines, and subsequently leading to unexpected in vivo findings. Given this, our study demonstrates that a successful targeting strategy, especially for DC-based subset targeting, requires coordinated interplay of the nanocarrier, the targeting ligand, and the spacer molecule. These findings pave the way to further optimize efficient and specific targeting of dendritic cells in vivo using antibody- or nanobody-functionalized nanocarriers for the use in cancer immunotherapy.

## Data Availability

Not applicable.

## Supplementary Materials

The following supporting information can be downloaded at: https://www.mdpi.com/article/10.3390/pharmaceutics14081614/s1, Table S1: Physicochemical characterization of PEGylated and antibody-modified nanocarriers with and without a murine protein corona; Figure S1: PEGylated nanocarrier uptake analyzed by flow cytometry; Figure S2: Validation of nanocarrier PEGylation; Figure S3: RAW264.7 cell uptake of PEGylated nanocarriers; Figure S4: A longer PEG chain reduces internalization by macrophages; Figure S5: Qualitative analysis of desorbed corona proteins; Figure S6: Most abundant proteins detected in mouse plasma; Figure S7: Amount of desorbed corona proteins; Figure S8: TEM visualization of PEGylated nanocarriers in cell culture medium.

## References

[1] Friedman A.D., Claypool S.E., Liu R. (2013). The smart targeting of nanoparticles. Curr. Pharm. Des..

[2] Rosenblum D., Joshi N., Tao W., Karp J.M., Peer D. (2018). Progress and challenges towards targeted delivery of cancer therapeutics. Nat. Commun..

[3] Wilhelm S., Tavares A.J., Dai Q., Ohta S., Audet J., Dvorak H.F., Chan W.C. (2016). Analysis of nanoparticle delivery to tumours. Nat. Rev. Mater..

[4] Ledford H. (2016). Bankruptcy filing worries developers of nanoparticle cancer drugs. Nat. News.

[5] He H., Liu L., Morin E.E., Liu M., Schwendeman A. (2019). Survey of clinical translation of cancer nanomedicines—Lessons learned from successes and failures. Acc. Chem. Res..

[6] Mahmoudi M. (2018). Debugging nano–bio interfaces: Systematic strategies to accelerate clinical translation of nanotechnologies. Trends Biotechnol..

[7] Horejs C. (2021). From lipids to lipid nanoparticles to mRNA vaccines. Nat. Rev. Mater..

[8] Salvati A., Pitek A.S., Monopoli M.P., Prapainop K., Bombelli F.B., Hristov D.R., Kelly P.M., Åberg C., Mahon E., Dawson K.A. (2013). Transferrin-functionalized nanoparticles lose their targeting capabilities when a biomolecule corona adsorbs on the surface. Nat. Nanotechnol..

[9] Cifuentes-Rius A., de Puig H., Kah J.C.Y., Borros S., Hamad-Schifferli K. (2013). Optimizing the properties of the protein corona surrounding nanoparticles for tuning payload release. ACS Nano.

[10] Ilinskaya A.N., Dobrovolskaia M.A. (2016). Understanding the immunogenicity and antigenicity of nanomaterials: Past, present and future. Toxicol. Appl. Pharmacol..

[11] Wang X.W., Zhong X.Y., Li J.X., Liu Z., Cheng L. (2021). Inorganic nanomaterials with rapid clearance for biomedical applications. Chem. Soc. Rev..

[12] Miao Z., Chen S., Xu C.Y., Ma Y., Qian H., Xu Y., Chen H., Wang X., He G., Lu Y. (2019). PEGylated rhenium nanoclusters: A degradable metal photothermal nanoagent for cancer therapy. Chem. Sci..

[13] Vonarbourg A., Passirani C., Saulnier P., Benoit J.-P. (2006). Parameters influencing the stealthiness of colloidal drug delivery systems. Biomaterials.

[14] Fam S.Y., Chee C.F., Yong C.Y., Ho K.L., Mariatulqabtiah A.R., Tan W.S. (2020). Stealth coating of nanoparticles in drug-delivery systems. Nanomaterials.

[15] Suk J.S., Xu Q., Kim N., Hanes J., Ensign L.M. (2016). PEGylation as a strategy for improving nanoparticle-based drug and gene delivery. Adv. Drug Deliv. Rev..

[16] Schöttler S., Becker G., Winzen S., Steinbach T., Mohr K., Landfester K., Mailänder V., Wurm F.R. (2016). Protein adsorption is required for stealth effect of poly (ethylene glycol)-and poly (phosphoester)-coated nanocarriers. Nat. Nanotechnol..

[17] Kozma G., Shimizu T., Ishida T., Szebeni J. (2020). Anti-PEG antibodies: Properties, formation and role in adverse immune reactions to PEGylated nano-biopharmaceuticals. Adv. Drug Deliv. Rev..

[18] Anselmo A.C., Mitragotri S. (2019). Nanoparticles in the clinic: An update. Bioeng. Transl. Med..

[19] Juan A., Cimas F.J., Bravo I., Pandiella A., Ocaña A., Alonso-Moreno C. (2020). Antibody Conjugation of Nanoparticles as Therapeutics for Breast Cancer Treatment. Int. J. Mol. Sci..

[20] Dolcetti R., López-Soto A., Dal Col J. (2020). Dendritic Cell-Based Immunotherapy in Solid and Haematologic Tumors. Front. Immunol..

[21] Calmeiro J., Carrascal M.A., Tavares A.R., Ferreira D.A., Gomes C., Falcão A., Cruz M.T., Neves B.M. (2020). Dendritic cell vaccines for cancer immunotherapy: The role of human conventional type 1 dendritic cells. Pharmaceutics.

[22] Collin M., Bigley V. (2018). Human dendritic cell subsets: An update. Immunology.

[23] Simon J., Fichter M., Kuhn G., Brückner M., Kappel C., Schunke J., Klaus T., Grabbe S., Landfester K., Mailänder V. (2022). Achieving dendritic cell subset-specific targeting in vivo by site-directed conjugation of targeting antibodies to nanocarriers. Nano Today.

[24] Johnston M.C., Scott C.J. (2018). Antibody conjugated nanoparticles as a novel form of antibody drug conjugate chemotherapy. Drug Discov. Today Technol..

[25] Stefanick J.F., Ashley J.D., Kiziltepe T., Bilgicer B. (2013). A systematic analysis of peptide linker length and liposomal polyethylene glycol coating on cellular uptake of peptide-targeted liposomes. ACS Nano.

[26] Jeong H.S., Sook Na K., Hwang H., Oh P.S., Hyun Kim D., Tae Lim S., Sohn M.H., Jeong H.J. (2014). Effect of space length of mannose ligand on uptake of mannosylated liposome in RAW 264.7 cells: In vitro and in vivo studies. J. Biomed. Mater. Res. Part A.

[27] Cruz L.J., Tacken P.J., Fokkink R., Figdor C.G. (2011). The influence of PEG chain length and targeting moiety on antibody-mediated delivery of nanoparticle vaccines to human dendritic cells. Biomaterials.

[28] Kapadia C.H., Tian S., Perry J.L., Luft J.C., DeSimone J.M. (2019). Role of linker length and antigen density in nanoparticle peptide vaccine. ACS Omega.

[29] Brückner M., Simon J., Landfester K., Mailänder V. (2021). The conjugation strategy affects antibody orientation and targeting properties of nanocarriers. Nanoscale.

[30] Tenzer S., Docter D., Rosfa S., Wlodarski A., Kuharev J., Rekik A., Knauer S.K., Bantz C., Nawroth T., Bier C. (2011). Nanoparticle Size Is a Critical Physicochemical Determinant of the Human Blood Plasma Corona: A Comprehensive Quantitative Proteomic Analysis. ACS Nano.

[31] Kokkinopoulou M., Simon J., Landfester K., Mailander V., Lieberwirth I. (2017). Visualization of the protein corona: Towards a biomolecular understanding of nanoparticle-cell-interactions. Nanoscale.

[32] Bradshaw R.A., Burlingame A.L., Carr S., Aebersold R. (2006). Reporting protein identification data: The next generation of guidelines. Mol. Cell. Proteom..

[33] Silva J.C., Gorenstein M.V., Li G.Z., Vissers J.P., Geromanos S.J. (2006). Absolute quantification of proteins by LCMSE: A virtue of parallel MS acquisition. Mol. Cell. Proteomics.

[34] Bros M., Montermann E., Cholaszczynska A., Reske-Kunz A.B. (2016). The phosphodiesterase 4 inhibitor roflumilast augments the Th17-promoting capability of dendritic cells by enhancing IL-23 production, and impairs their T cell stimulatory activity due to elevated IL-10. Int. Immunopharmacol..

[35] Passlick D., Piradashvili K., Bamberger D., Li M., Jiang S., Strand D., Wich P.R., Landfester K., Bros M., Grabbe S. (2018). Delivering all in one: Antigen-nanocapsule loaded with dual adjuvant yields superadditive effects by DC-directed T cell stimulation. J. Control. Release.

[36] Caracciolo G., Palchetti S., Colapicchioni V., Digiacomo L., Pozzi D., Capriotti A.L., La Barbera G., Laganà A. (2015). Stealth effect of biomolecular corona on nanoparticle uptake by immune cells. Langmuir.

[37] Li M., Jiang S., Simon J., Paßlick D., Frey M.-L., Wagner M., Mailänder V., Crespy D., Landfester K. (2021). Brush Conformation of Polyethylene Glycol Determines the Stealth Effect of Nanocarriers in the Low Protein Adsorption Regime. Nano Lett..

[38] Li S.-D., Huang L. (2010). Stealth nanoparticles: High density but sheddable PEG is a key for tumor targeting. J. Control. Release Off. J. Control. Release Soc..

[39] Owens D.E., Peppas N.A. (2006). Opsonization, biodistribution, and pharmacokinetics of polymeric nanoparticles. Int. J. Pharm..

[40] Salmaso S., Caliceti P. (2013). Stealth properties to improve therapeutic efficacy of drug nanocarriers. J. Drug Deliv..

[41] Müller L.K., Simon J., Rosenauer C., Mailänder V., Morsbach S., Landfester K. (2018). The transferability from animal models to humans: Challenges regarding aggregation and protein corona formation of nanoparticles. Biomacromolecules.

[42] Ricklin D., Hajishengallis G., Yang K., Lambris J.D. (2010). Complement: A key system for immune surveillance and homeostasis. Nat. Immunol..

[43] Moghimi S.M., Szebeni J. (2003). Stealth liposomes and long circulating nanoparticles: Critical issues in pharmacokinetics, opsonization and protein-binding properties. Prog. Lipid Res..

[44] Walkey C.D., Olsen J.B., Guo H., Emili A., Chan W.C. (2012). Nanoparticle size and surface chemistry determine serum protein adsorption and macrophage uptake. J. Am. Chem. Soc..

[45] Cruz L.J., Rosalia R.A., Kleinovink J.W., Rueda F., Löwik C.W., Ossendorp F. (2014). Targeting nanoparticles to CD40, DEC-205 or CD11c molecules on dendritic cells for efficient CD8+ T cell response: A comparative study. J. Control. Release.

[46] Ammon C., Meyer S., Schwarzfischer L., Krause S., Andreesen R., Kreutz M. (2000). Comparative analysis of integrin expression on monocyte—Derived macrophages and monocyte-derived dendritic cells. Immunology.

[47] Berry J.D., Licea A., Popkov M., Cortez X., Fuller R., Elia M., Kerwin L., Kubitz D., Barbas C.F. (2003). Rapid monoclonal antibody generation via dendritic cell targeting in vivo. Hybrid. Hybridomics.

[48] Banchereau J., Briere F., Caux C., Davoust J., Lebecque S., Liu Y.-J., Pulendran B., Palucka K. (2000). Immunobiology of dendritic cells. Annu. Rev. Immunol..

