# Supplementary material for: PEG Spacer Length Substantially Affects Antibody-Based Nanocarrier Targeting of Dendritic Cell Subsets
Source: Pharmaceutics. 2022 Aug 2;14(8):1614. doi: 10.3390/pharmaceutics14081614 (PMC9414227; doi:10.3390/pharmaceutics14081614)
Supplement: Supplementary file 1 [file pharmaceutics-14-01614-s001.zip › pharmaceutics-1821626-supplementary.pdf]

Supporting Information

PEG Spacer Length Substantially Affects Antibody-Based Nanocarrier Targeting of Dendritic Cell Subsets

Maximilian Brückner [1,2,†], Michael Fichter [1,2,†], Richard da Costa Marques [1,2], Katharina Landfester [2] and Volker Mailänder [1,2,*]

[1] Department of Dermatology, University Medical Center of the Johannes Gutenberg University Mainz, Langenbeckstr. 1, 55131 Mainz, Germany; brueckner@mpip-mainz.mpg.de (M.B.); fichter@uni-mainz.de (M.F.); dacostamarques@mpip-mainz.mpg.de (R.d.C.M.)
[2] Max Planck Institute for Polymer Research, Ackermannweg 10, 55128 Mainz, Germany; landfester@mpip-mainz.mpg.de
* Correspondence: volker.mailaender@unimedizin-mainz.de
† These authors contributed equally to this work.

Supporting Figures and Tables

Table S1. Physicochemical characterization of PEGylated and antibody-modified nanocarriers with and without a murine protein corona. The average size (diameter in nm) and size distribution (PDI) of the nanocarriers was determined by multi-angle dynamic light scattering, and the zeta (ξ) potential was measured.

	Without a Protein Corona		Pre-Incubated with Mouse Plasma	
	Diameter in nm with PDI	ξ-Potential in mV with SD	Diameter in nm with PDI	ξ-Potential in mV with SD
**Pristine nanocarriers**				
**mgHES**	258 (0.156)	−0.90 (0.041)	208 (0.108)	−7.94 (0.601)
**PEGylated nanocarriers**				
**0.65 kDa DBCO**	232 (0.176)	−9.18 (0.154)	234 (0.158)	−11.9 (0.458)
**2 kDa MeO**	220 (0.126)	−4.90 (0.466)	218 (0.212)	−5.47 (1.670)
**2 kDa DBCO**	220 (0.107)	−5.02 (0.899)	220 (0.140)	−7.72 (0.893)
**5 kDa MeO**	202 (0.086)	−3.65 (0.574)	258 (0.211)	−4.95 (0.559)
**5 kDa DBCO**	198 (0.141)	−3.75 (0.665)	244 (0.214)	−7.29 (1.838)
**Antibody-modified nanocarriers**				
**0.65 kDa CD11c**	220 (0.235)	−6.28 (0.467)	206 (0.147)	−6.65 (0.120)
**0.65 kDa Isotype**	200 (0.186)	−4.98 (0.937)	168 (0.095)	−5.60 (0.675)

**2 kDa CD11c**	262 (0.217)	1.19 (0.0781)	170 (0.098)	−3.69 (0.0961)
**2 kDa Isotype**	196 (0.135)	0.165 (0.658)	200 (0.186)	−5.98 (0.411)
**5 kDa CD11c**	206 (0.210)	4.13 (0.137)	212 (0.117)	−4.81 (0.131)
**5 kDa Isotype**	218 (0.154)	5.05 (0.182)	168 (0.169)	−5.17 (0.192)

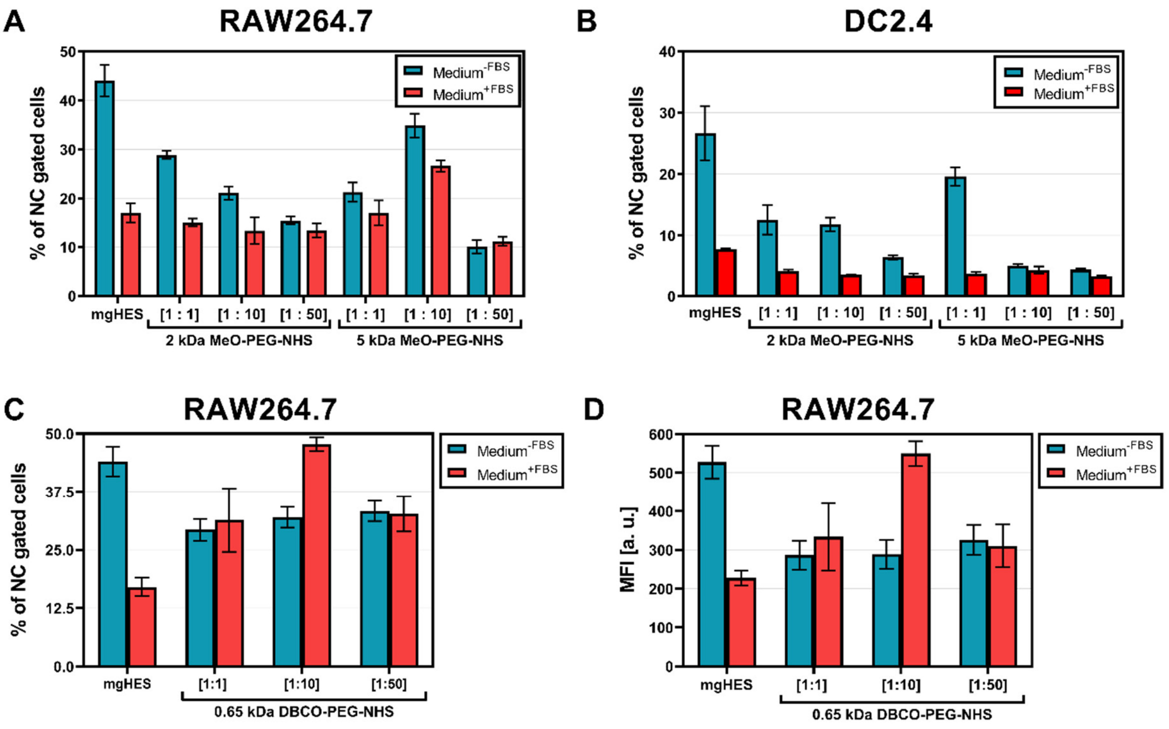

Figure S1. PEGylated nanocarrier uptake analyzed by flow cytometry. Cell uptake of pristine nanocarriers (mgHES) and PEGylated samples with 7.5 µg mL^{-1} in medium with (red bars) or without (blue bars) FBS. PEGylation with the 2 and 5 kDa NHS-PEG-MeO linkers was performed with three molar reaction ratios (1:1, 1:10, 1:50) in either the RAW264.7 (**A**) or the DC2.4 (**B**) cell lines and analyzes by flow cytometry (percentage of nanocarrier gated cells). PEGylation with the 0.65 kDa NHS-PEG-DBCO linker was performed with the same three molar reaction ratios in the RAW264.7 cell line either analyzed as the percentage of nanocarrier gated cells (**C**) or as the median fluorescence intensity (**D**). Auto fluorescence is subtracted from each data set. Values are given as mean ± SD (n = 3). Only viable cells are gated.

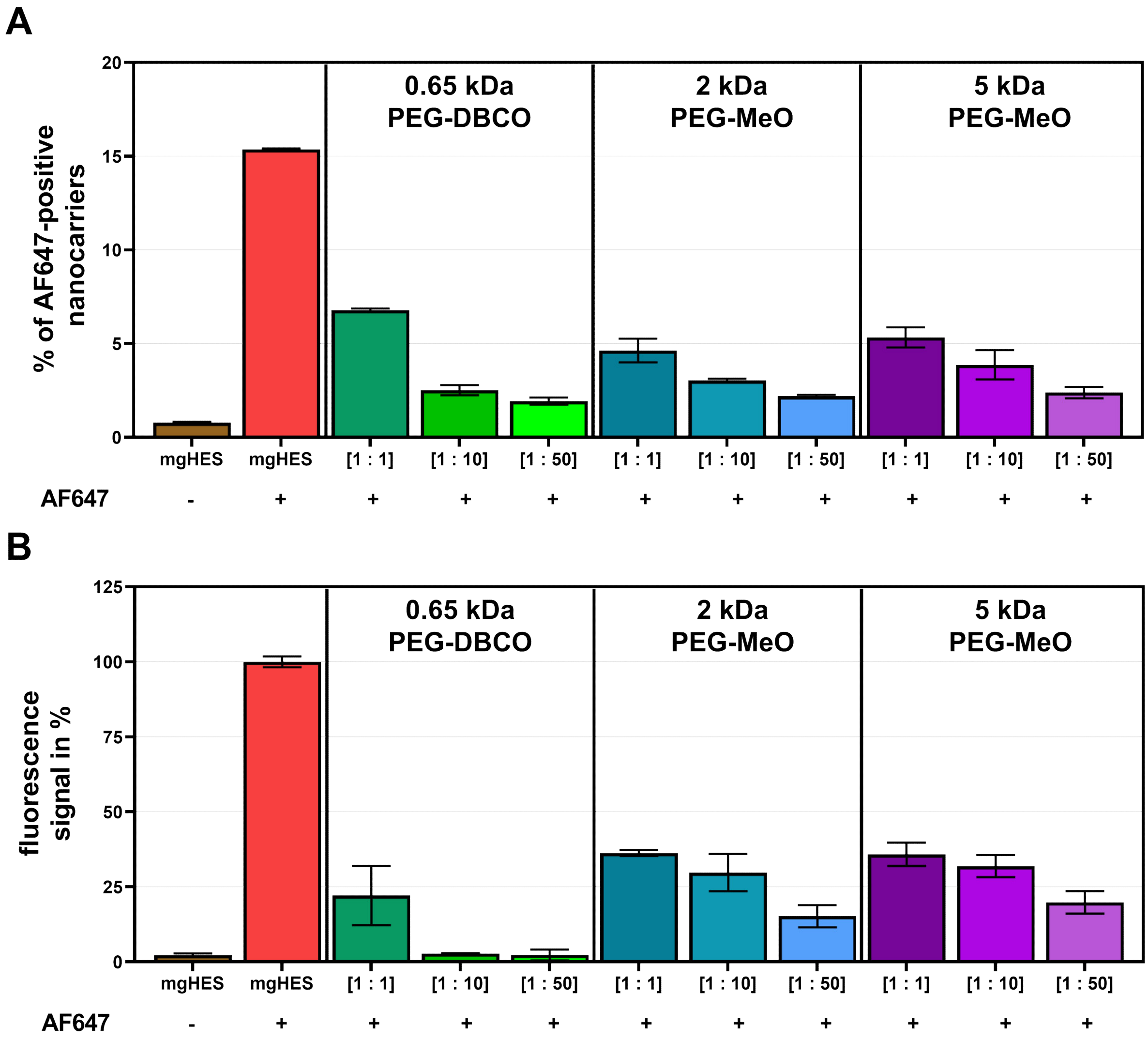

Figure S2. Validation of nanocarrier PEGylation. The surface-modification of the nanocarriers with PEG was analyzed by flow cytometry (**A**) and plate reader (**B**). For this investigation, the pristine nanocarriers (mgHES) and the samples with different PEG ratios (1:1, 1:10, 1:50) were incubated with an Alexa Fluor 647 NHS-Ester followed by washing to remove unconjugated moieties. The fluorescence was then detected by the two devices mentioned above.

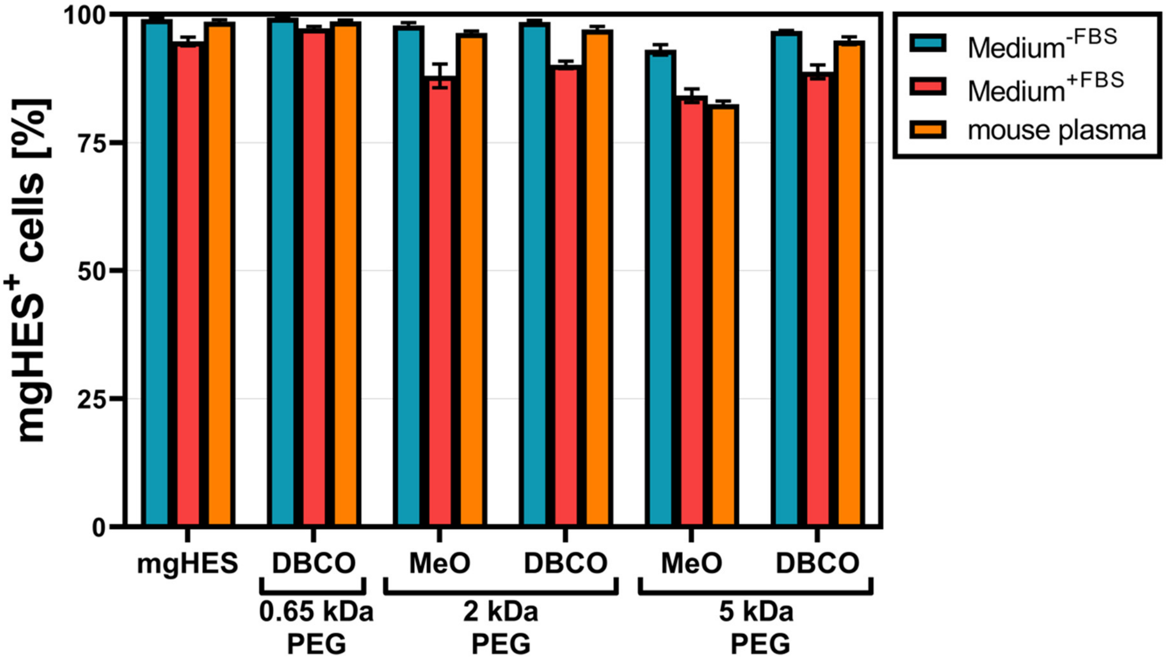

Figure S3. RAW264.7 cell uptake of PEGylated nanocarriers. Nanocarriers were incubated with RAW264.7 cells in either medium with or without FBS, or pre-incubated with mouse plasma with a sample concentration of 75 µg mL^{-1} for 2 h at 37 °C. Autofluorescence was subtracted from each data set. Only viable cells were analyzed. Values are given as mean ± SD (*n* = 3).

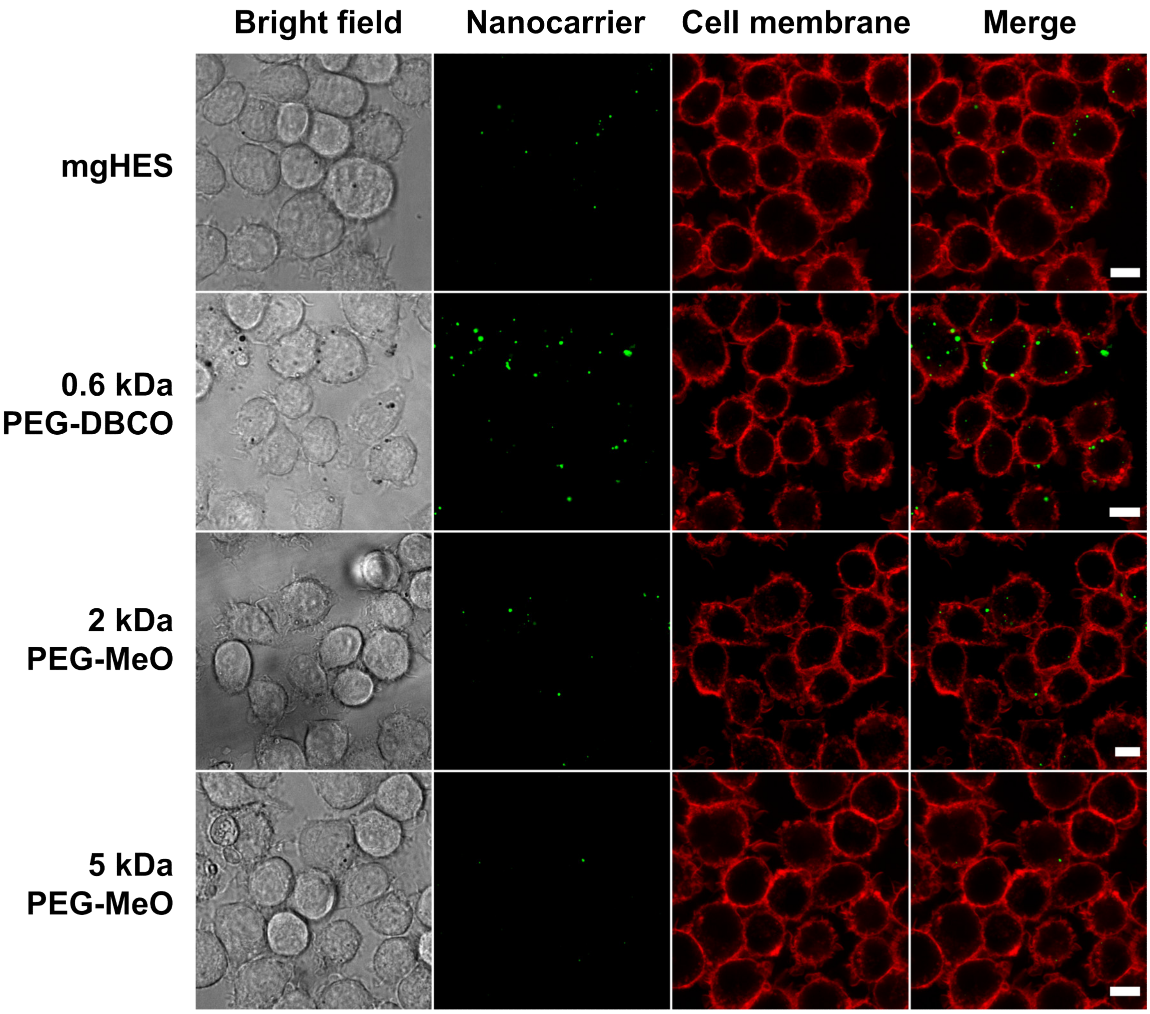

Figure S4. A longer PEG chain reduces internalization by macrophages. Nanocarriers were incubated with the RAW264.7 cells for two hours at 37 °C with a sample concentration of 75 μg mL^{-1} in DMEM medium with 10% FBS. Live cell images are shown. All scale bars represent 10 μm.

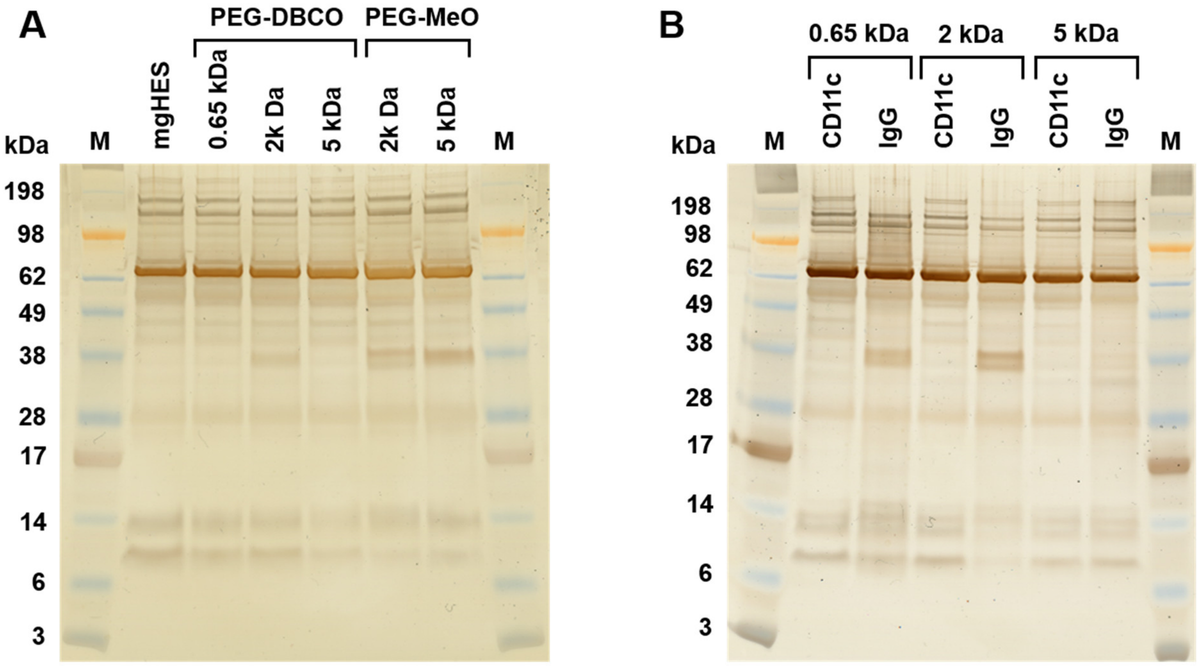

Figure S5. Qualitative analysis of desorbed corona proteins. SDS-PAGE of PEGylated (**A**) and targeting (**B**) samples is shown. Following 1 h incubation in mouse plasma at 37 °C, murine proteins were desorbed and separated from the nanocarrier. The protein amount was quantified and 2 µg were used for the gel. The SDS-PAGE was run for 1 h at 120 V followed by silver staining.

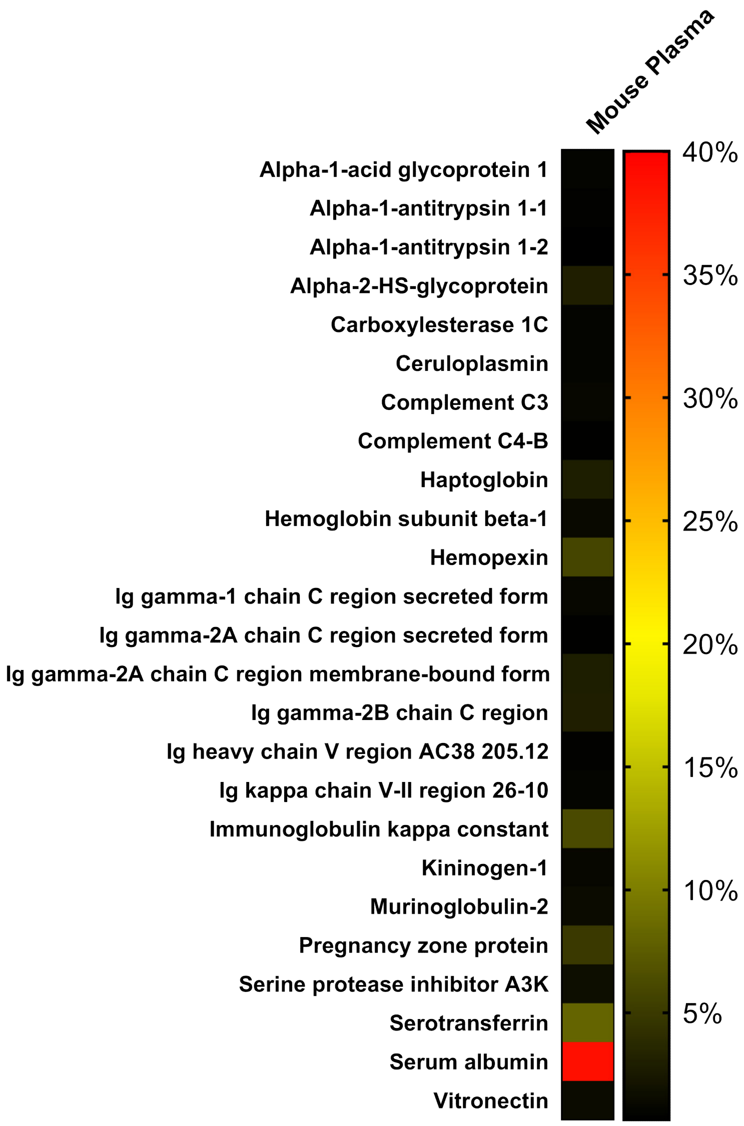

Figure S6. Most abundant proteins detected in mouse plasma. Heat map of the top 25 most abundant proteins in mouse plasma are shown. The values are expressed in percentage based on the total amount of all identified proteins.

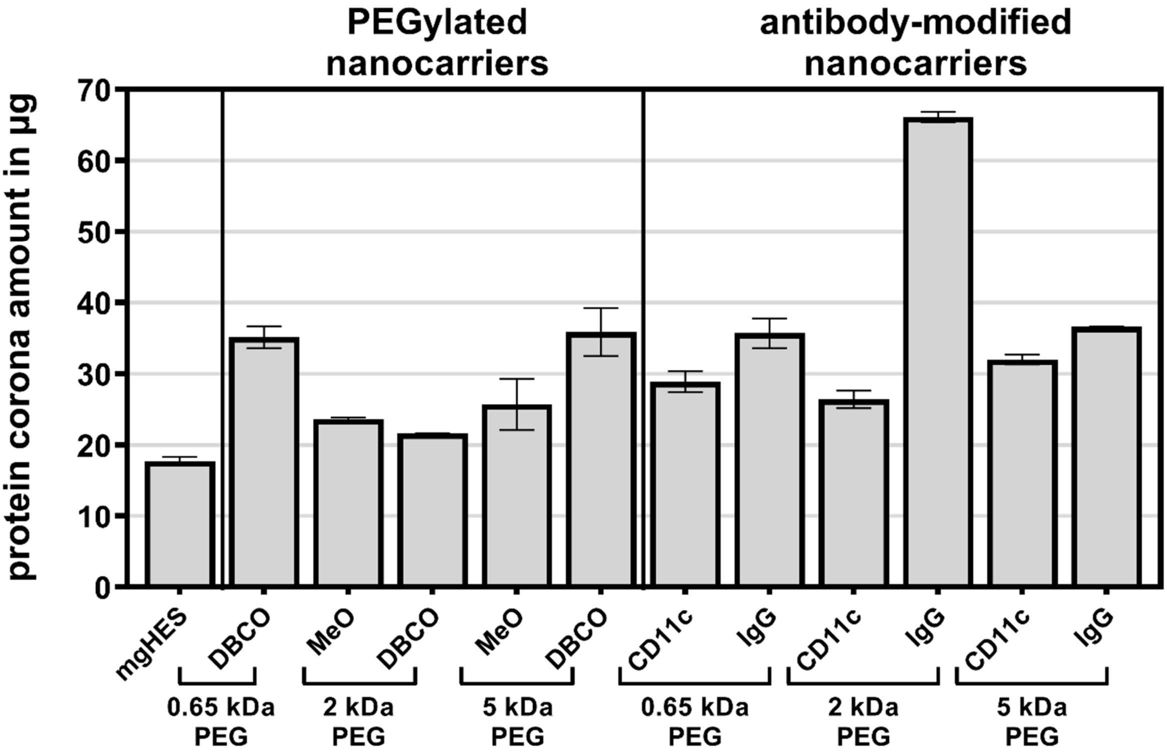

Figure S7. Amount of desorbed corona proteins. After the pre-incubation with mouse plasma, the protein corona was desorbed from the nanocarriers by desorption buffer. The protein amount was determined by Pierce™ 660nm Protein Assay. Data is shown as mean ± standard deviation (*n* = 2).

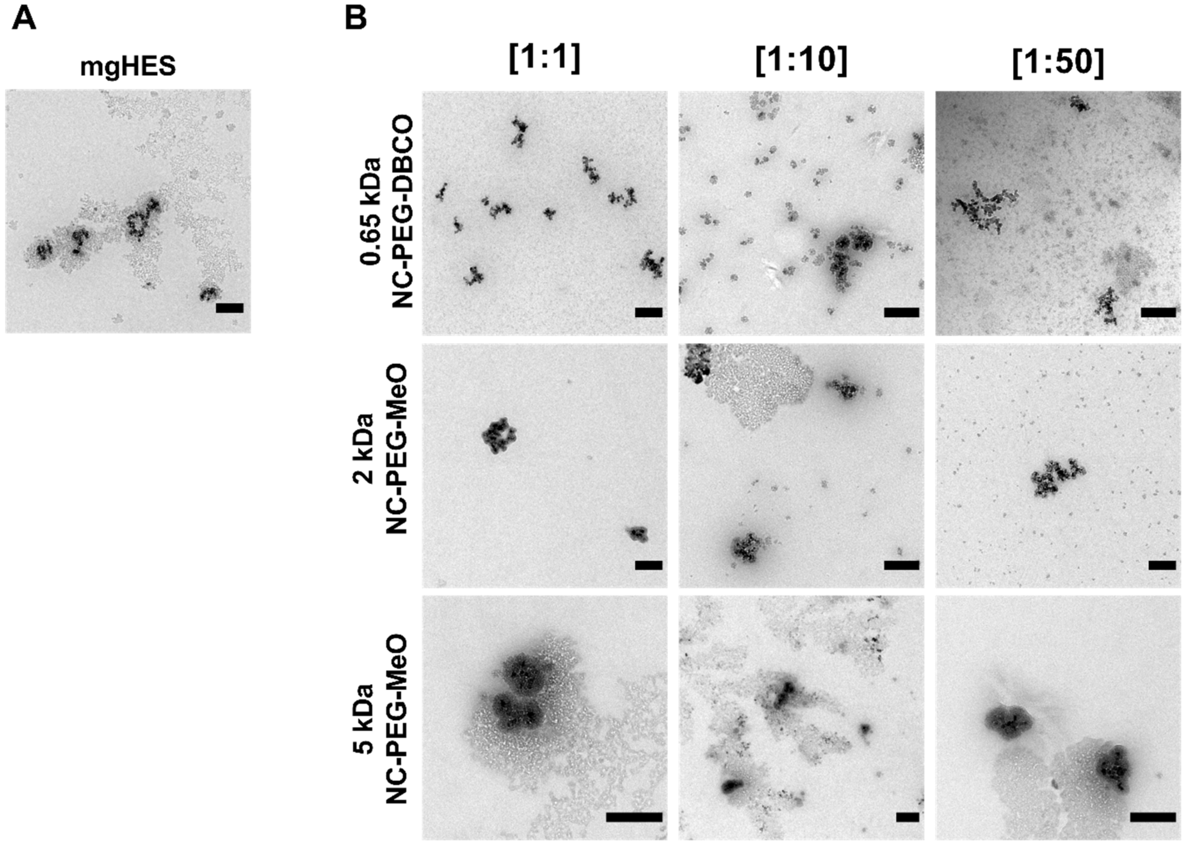

Figure S8. TEM visualization of PEGylated nanocarriers in cell culture medium. Pristine nanocarriers (**A**) and PEGylated nanocarriers (**B**) were diluted to a concentration of 75 µg mL^{-1} in IMDM medium containing 5% FBS and were imaged by TEM. The PEGylation was performed with three different molecular weight linkers (0.65 kDa NHS-PEG-DBCO, 2 kDa and 5 kDa NHS-PEG-MeO) and three nanocarrier to linker molar reaction ratios (1:1, 1:10, 1:50). Grey clouds are most likely salt contaminations from the cell culture medium. The scale bars represent 200 nm.
